# Maternal periodontitis as a risk factor for preterm birth: A cross-sectional study

**DOI:** 10.34172/joddd.40860

**Published:** 2024-03-29

**Authors:** Syed Imran Gilani, Aiman Niaz, Saira Afridi

**Affiliations:** Department of Community & Preventive Dentistry, Sardar Begum Dental College, Peshawar, Pakistan

**Keywords:** Dental caries, Dentists, Infections, Maternal mortality, Oral health, Oral, Periodontics, Periodontitis, Pregnancy, Premature birth

## Abstract

**Background.:**

Preterm birth is a heterogeneous condition with multiple underlying causes, and periodontal diseases are one of them. Approximately 900000 preterm births are reported in Pakistan each year. Oral infections such as periodontitis during pregnancy are associated with adverse pregnancy outcomes such as low birth weight and preterm births. However, different studies have reported contradictory findings. We conducted a cross-sectional study to assess the association of preterm birth with oral infection in pregnancy.

**Methods.:**

We conducted a cross-sectional analytical study on 400 postpartum pregnant women in Khyber Teaching Hospital, Peshawar. Only women within the age bracket of 18‒40 years were recruited. Data were collected by an interview-based structured questionnaire. The extent and severity index were used to assess the periodontal health of participants. Frequency tables were generated, and the chi-squared test was used to determine associations between different categorical variables.

**Results.:**

The mean age of the participants was 25.8±4.9 years. Approximately 87.5% of the women had generalized periodontitis. Approximately 68% of mothers had moderate severity of periodontitis. The extent index showed no notable difference between the preterm and full-term birth groups. In contrast, the severity index displayed a statistically significant difference between the preterm and full-term birth groups.

**Conclusion.:**

The majority of women had generalized periodontitis. The severity index demonstrated a significant association between maternal periodontitis and preterm births. There was no association between the age of mothers and preterm births. Complications in pregnancy were not associated with preterm births.

## Introduction

 Preterm birth is a public health concern with significant psychological and financial implications.^1^ Regardless of improvements in obstetric care and neonatal science, the count of preterm births is not decreasing, and no medicine seems to stop premature births.^2,3^ Approximately half of preterm births are associated with intrauterine infections.^4^ Oral infections such as periodontal diseases are a potential risk factor for preterm births.^5^

 The World Health Organization (WHO) stated that delivery before 37 weeks ( < 259 days) from the last date of the menstrual cycle is termed preterm birth.^2^ It has a global prevalence of 11.1% and is one of the leading causes of neonatal fatality in Western and developing countries.^4^

 Approximately 15 million preterm births are reported each year, resulting in one million deaths due to complications.^2^ The incidence is higher in Pakistan, Mauritania, and Indonesia.^1^

 UNICEF reported that Pakistan is second among the top ten countries, accounting for two-thirds of all deaths resulting from premature birth complications. Approximately 900 000 preterm births are reported in Pakistan annually, and more than 10% of these premature babies die due to complications.^6^

 Preterm babies are likely to suffer from several critical health problems that can adversely impact the lives of newborns, parents, family members, and society members.^2^ Poor health outcomes include chronic lung diseases, gastroesophageal diseases, cardiovascular disorders, compromised immunity, and poor motor, sensory, and cognitive skills. Premature newborns mostly require a neonatal intensive care unit for monitoring their vital signs,^3^ subsequently increasing the economic burden in hospitals. If not appropriately managed, these newborns have a high risk of experiencing lifelong disability.^2^

 Preterm birth is associated with systemic infections, e.g., syphilis, genitourinary tract infections, chlamydia, and pneumonia. Infections during pregnancy may endanger the fetus’s life by activating the innate defense system, eliciting the production of inflammatory cytokines and prostaglandins, which may result in preterm labor.^7^

 Oral infections in pregnancy are a significant healthcare issue due to their pervasiveness worldwide and adverse outcomes on the mother and newborn.^8^ A decline in periodontal health status is reported in pregnant women because of elevated levels of sex hormones in blood plasma.^9^ The oral health of pregnant women is at risk for disease for various reasons, such as a sudden shift in sex hormone levels causing inflammation and low immunity during pregnancy.^10^ In gestation, progesterone is 30 times higher, and estrogen is 10 times higher than in menstrual cycles.^11^ Moreover, pregnant women are more likely to consume carbohydrate-rich and cariogenic food.^10^ These circumstances provide a favorable environment for bacteria to flourish and cause oral infections.^12^

 Periodontitis is an inflammatory disease characterized by the destruction of alveolar bone, periodontal ligaments, and other structures surrounding the tooth.^13^ One in every five pregnant women has periodontitis.^14^ Approximately 40% of pregnant women exhibit clinical signs of periodontal diseases.^8^ Research has shown that pregnant women with periodontal diseases are seven times more prone to deliver premature babies.^10^

 The possible mechanism of oral infections triggering preterm delivery is based on two hypotheses. Pathogenic bacteria are transmitted from the oral cavity to the fetoplacental unit via the hematogenous route,^5^ liberating IL-1, IL-6, IL-8, TNF-a, and PGE2 in the amniotic cavity, whose anti-inflammatory action may cause preterm birth.^15^ The second hypothesis suggests that inflammatory mediators produced due to periodontal disease could intensify intrauterine inflammation and lead to premature delivery.^5^

 Research demonstrates an association between maternal periodontal diseases and complications in pregnancy, such as low birth weight and preterm births.^16^ Periodontitis progresses due to an immune response to the bacterial biofilm in the oral cavity.^4^ The inflamed tissues of the periodontium act as a reservoir for pathogenic microbes, inflammatory mediators, and endotoxins.^7^ The microbes triggering periodontitis are not only present in sulcular fluid, subgingival plaque, supragingival plaque, and other tooth-supporting tissue but also circulate and spread to the amniotic cavity, fetus, vagina, umbilical cord, and maternal serum. In addition, endotoxins derived from these pathogenic microbes can enter the placenta and trigger the production of PGE2 and IL-1β in trophoblastic and chorioamniotic cells, which weaken the uterine membrane and spontaneous uterine contractions, resulting in preterm birth.^14^

 The association of periodontitis and preterm birth has been the subject of many clinical trials and epidemiological studies over the past two decades. However, the findings have been inconsistent.^17,18^ Considering the global burden of preterm births, addressing this public health concern is a top priority. By generating evidence regarding the effect of periodontitis on preterm births, relevant policies could be created to promote healthy pregnancies and better access to dental care. The present study was conducted to evaluate the association between preterm birth and periodontitis in pregnant women in a tertiary care hospital in Peshawar.

## Methods

 This analytical cross-sectional study enrolled 400 postpartum pregnant women who visited the Gynecology Ward of Khyber Teaching Hospital, Peshawar, for delivery. The ethical approval was obtained from the Ethics Committee of Gandhara University, Peshawar. The sample size was computed on a 50% proportion of preterm births with a 95% confidence interval with a statistical power of 80%. We collected data for this study from June 2022 to October 2022. The purpose of the study was explained to the participants in detail, and written informed consent was obtained. An equal number of postpartum women with preterm births and full-term births was recruited in this study. Women aged 18‒40 years were recruited. Mothers with < 6 teeth and a history of systemic illness, e.g., diabetes mellitus or a history of preterm birth, were excluded from this study. In the present study, periodontal disease was the independent variable, while preterm birth was the dependent variable. Initially, a pilot study was performed on 30 participants. After making the required modifications, the process of data collection was initiated. Maternity notes were used to gather demographic information and information regarding medical history during pregnancy. Clinical examinations were conducted to assess the extent and severity index. Three dental practitioners were trained and calibrated for the extent and severity index. A periodontal probe was used to determine the percentage of sites affected by attachment loss. Twenty-eight sites comprising 14 sites on the upper arch and 14 at the contralateral half of the lower arch were measured. The sites assessed were mesiobuccal, interproximal, and midbuccal of all teeth except molars and mesiobuccal, interproximal, and midbuccal areas of mesial root of molars. The criteria for categorization of the extent and severity of periodontal disease are given in Tables 1 and 2.^19^ SPSS 20 was used to analyze data with a 95% confidence interval, and statistical significance was set at *P* < 0.05. The chi-squared test was used to determine the association between different categorical variables. Frequency tables and percentages were generated for the data. Possible confounding factors, such as complications during pregnancy, eclampsia, low amniotic fluid, and bleeding, were documented. A layered variable analysis on SPPS 20 was conducted to rule out their influence on the results.

**Table 1:** Criteria for the extent of periodontal disease

**Condition**	**Criteria**
Localized condition	Equal to or less than 30% of sites involved
Generalized condition	Greater than 30% of sites involved

**Table 2:** Criteria for the severity of periodontal disease

**Condition**	**Criteria**
Mild periodontitis	More than 1 but less than 3 mm CAL
Moderate periodontitis	Equal to 3mm but less than 5 mm CAL, possible accompanied by an increase in tooth mobility and furcation involvement.
Severe periodontitis	5mm of greater CAL, accompanied by tooth mobility, furcation involvement and muco-gingival defects.

## Results

 The present study was conducted on 400 pregnant women with a mean age of 25.8 ± 4.9 years. The minimum age of the participants was 18 years, and the maximum was 40. The findings using the extent index and its association with complications in pregnancy are presented in Tables 3 and 4. Tables 5 and 6 present the periodontal status of pregnant women using the severity index and its association with complications in pregnancy.

**Table 3:** Results of the extent index

**Extent index categories**	**Preterm, No. (%)**	**Full term, No. (%)**	**Total, No. (%)**
Normal periodontitis	6 (3.0)	6 (3.0)	12 (3.0)
Localized periodontitis	14 (7.0)	24 (12.0)	38 (9.5)
Generalized periodontitis	180 (90.0)	170 (85.0)	350 (87.5)

*P* value = 0.233

**Table 4:** Extent index categories and their association with complications in pregnancy

**Complications during pregnancy**	**Extent index categories**	**Preterm, No. (%)**	**Full term, No. (%)**	**Total, No. (%)**	***P*** ** value**
No complications	Normal	4 (2.6)	6 (3.2)	10 (2.9)	0.260
	Localized periodontitis	8 (5.1)	18 (9.7)	26 (7.6)	
	Generalized periodontitis	144 (92.3)	162 (87.1)	306 (89.5)	
Hypertension	Localized periodontitis	2 (14.3)	4 (40.0)	6 (25.0)	0.151
	Generalized periodontitis	12 (85.7)	6 (60.0)	18 (75.0)	
	Total	14 (100.0)	10 (100.0)	24 (100.0)	
Eclampsia	Normal	2 (10)	0 (0)	2 (9.1)	0.783
	Localized periodontitis	2 (10.0)	0 (0)	2 (9.1)	
	Generalized periodontitis	16 (80.0)	2 (100.0)	18 (81.8)	
Low amniotic fluid	Generalized periodontitis	2 (100.0)		2 (100.0)	
	Total	2 (100)		2 (100.0)	
Cord around the neck	Localized periodontitis	2 (33.3)	2 (100)	4 (50)	0.102
	Generalized periodontitis	4 (66.7)	0 (0)	4 (50.0)	
	Total	6 (100)	2 (100)	8 (100)	
Bleeding	Generalized periodontitis	2 (100.0)		2 (100.0)	
	Total	2 (100.0)		2 (100.0)	

**Table 5:** Results of the severity index

**Severity index categories**	**Preterm births, No. (%)**	**Full term births, No. (%)**	**Total,** **No. (%)**	***P*** ** value**
No disease	18 (9.0)	28 (14.0)	46 (11.5)	0.000
Mild	6 (3.0)	54 (27.0)	60 (15.0)	
Moderate	162 (81.0)	110 (55.0)	272 (68.0)	
Severe	14 (7.0)	8 (4.0)	22 (5.5)	
Total	200 (100.0)	200 (100.0)	400 (100.0)	

**Table 6:** Severity index categories and their association with complications in pregnancy

**Complications during pregnancy**	**Severity index categories**	**Preterm births, No. (%)**	**Full term births, No. (%)**	**Total, No. (%)**	***P*** ** value**
No complications	No disease	12 (7.7)	26 (14.0)	38 (11.1)	0.000
	Mild	4 (2.6)	54 (29.0)	58 (17)	
	Moderate	128 (82.1)	98 (52.7)	226 (66.1)	
	Severe	12 (7.7)	8 (4.3)	20 (5.8)	
	Total	156 (100)	186 (100)	342 (100)	
Hypertension	No disease	4 (28.6)	0 (0)	4 (16.7)	0.057
	Moderate	8 (57.1)	10 (100)	18 (75)	
	Severe	2 (14.3)	0 (0)	2 (8.3)	
	Total	14 (100)	10 (100)	24 (100)	
Eclampsia	No disease	2 (10)	2 (100)	4 (18.2)	0.007
	Mild	2 (10)	0 (0)	2 (9.1)	
	Moderate	16 (80)	0 (0)	16 (72.7)	
	Total	20 (100)	2 (100)	22 (100)	
Low amniotic fluid	Moderate	2 (100)		2 (100)	N/A
	Total	2 (100)		2 (100)	
Cord around neck	Moderate	6 (100)	2 (100)	8 (100)	N/A
	Total	6 (100)	2 (100)	8 (100)	
Bleeding	Moderate	2 (100)		2 (100)	N/A
	Total	2 (100)		2 (100)	

## Discussion

 We conducted an observational cross-sectional study to determine the association between preterm births and periodontitis using the extent and severity indexes. The mean age of the women was 25.88 (SD = 4.915) years. There was no significant association between the age of the participants and preterm births. A previous study stated that women in their early twenties had lower chances, while women above the age of 30 and teenage girls had a high risk of premature delivery.^20^

 The data generated regarding periodontal diseases are controversial due to different definitions of periodontitis and preterm births, sampling techniques, type of periodontal index used, differences in the geographic area, and ethnicity of participants.^21,22^

 In the present study, approximately 87.5% of the pregnant women had generalized periodontitis. There was no statistically significant association between the full-term and preterm birth groups. Approximately 68% of participants exhibited moderate severity of periodontitis. Unlike the extent index, the severity index showed a statistically significant difference between the preterm and full-term birth groups. A case‒control study in 2017 stated that the extent and severity levels of periodontitis were not significantly different between mothers of preterm and full-term births.^23^

 Offenbacher et al^22^ reported that women with periodontitis had a 7.5-fold greater chance of preterm birth than women with no periodontitis.A case‒control study in Africa assessed the association between periodontitis and preterm births using the Community Periodontal Index. The study stated that women with periodontitis had a two-fold greater chance of delivering preterm babies than women who had no periodontitis (odds ratio = 2.05).^24^ A retrospective unmatched case‒control study in Rwanda concluded that women with periodontitis had six-fold higher chances of premature delivery than periodontally healthy women (odds ratio: 6.360).^25^ In 2020, a hospital-based cross-sectional study using postpartum full-mouth periodontal examination reported that women with severe periodontitis had 3.46-fold higher odds of preterm births than non-periodontitis pregnant women.^22^

 In contrast to the above studies, many studies have reported no association between preterm birth and periodontitis. A descriptive correlational study conducted in Iran in 2017 concluded that there was no association of maternal periodontitis with pocket depth, bleeding on probing and clinical attachment loss and preterm births.^26^

 As per 5 longitudinal studies, there was no difference between women with periodontitis and those without periodontitis concerning preterm delivery.^27^

 Previous literature stated that complications in pregnancy, such as eclampsia, bleeding during pregnancy, hypertension, and low amniotic fluid (oligohydramnios), were associated with preterm births.^28^ We used layered variable analysis to assess the association of periodontal diseases and preterm births to rule out the impact of confounding variables on the results.

 The difference between the two groups was also not significant when computed with complications in pregnancy. The present study found no association between periodontal infection and eclampsia in pregnancy. A study in the United States reported that women with severe periodontitis or periodontal infection progression in pregnancy had a greater chance of preeclampsia (odds ratio = 2.40).^27^

 The healthcare system of Pakistan is in constant crisis, and preterm births contribute to the economic burden on hospitals.^28^ A study conducted at Fatima Jinnah Medical College, Lahore, in 2014 revealed a significant association between periodontitis in pregnancy and preterm births.^20^ A study in the Civil Hospital of Karachi assessed x-rays of postpartum women for chronic apical periodontitis. The study stated that the periapical index score was significantly associated with the birth type.^29^

 The present study was conducted at a single institute with a small sample size, which limits the generalizability of the findings. The present study used a cross-sectional design. For future studies, a comprehensive randomized clinical trial is recommended.

## Conclusion

 Within the limitations of this study, it can be concluded that the majority of pregnant women examined in this study had generalized periodontitis. The extent of periodontal disease had no association with preterm births, while the association of the severity of periodontal disease was statistically significant. There was no statistically significant difference in terms of complications in pregnancy between the preterm and full-term groups.

## Competing Interests

 The authors declare no conflicts of interest.

## Ethical Approval

 Ethical approval was obtained from the Ethics Committee, Gandhara University Peshawar.

## Funding

 The funding for this research was entirely self-funded by the authors.
